# Understanding immune microenvironment alterations in the brain to improve the diagnosis and treatment of diverse brain diseases

**DOI:** 10.1186/s12964-024-01509-w

**Published:** 2024-02-17

**Authors:** Xiaotong Xu, Yi Han, Binlong Zhang, Quanzhong Ren, Juan Ma, Sijin Liu

**Affiliations:** 1 State Key Laboratory of Environmental Chemistry and Ecotoxicology, Research Center for Eco-Environmental Sciences, Chinese Academy of Sciences, Beijing, 100085 People’s Republic of China; 2 University of Chinese Academy of Sciences, Beijing, 100049 People’s Republic of China; 3 Guang’an Men Hospital, China Academy of Chinese Medical Sciences, Beijing, 100053 People’s Republic of China; 4 JST Sarcopenia Research Centre, National Center for Orthopaedics, Beijing Research Institute of Traumatology and Orthopaedics, Beijing Jishuitan Hospital, Capital Medical University, Beijing, 100035 People’s Republic of China; 5 Medical Science and Technology Innovation Center, Shandong First Medical University & Shandong Academy of Medical Sciences, Jinan, Shandong 250117 People’s Republic of China

**Keywords:** Brain disease, Immune microenvironment, Cerebrospinal fluid, Disease diagnosis and therapeutics

## Abstract

Abnormal inflammatory states in the brain are associated with a variety of brain diseases. The dynamic changes in the number and function of immune cells in cerebrospinal fluid (CSF) are advantageous for the early prediction and diagnosis of immune diseases affecting the brain. The aggregated factors and cells in inflamed CSF may represent candidate targets for therapy. The physiological barriers in the brain, such as the blood‒brain barrier (BBB), establish a stable environment for the distribution of resident immune cells. However, the underlying mechanism by which peripheral immune cells migrate into the brain and their role in maintaining immune homeostasis in CSF are still unclear. To advance our understanding of the causal link between brain diseases and immune cell status, we investigated the characteristics of immune cell changes in CSF and the molecular mechanisms involved in common brain diseases. Furthermore, we summarized the diagnostic and treatment methods for brain diseases in which immune cells and related cytokines in CSF are used as targets. Further investigations of the new immune cell subtypes and their contributions to the development of brain diseases are needed to improve diagnostic specificity and therapy.

## Background

It was previously believed that the brain was an “immune privileged” site under the protection of the blood‒brain barrier (BBB). However, with the progressive understanding of brain immunity and the rediscovery of meningeal lymphatic vessels [1], the relationships between immune cells and various brain disorders, such as aging, neurodegenerative diseases and multiple sclerosis (MS), have received increasing attention [2, 3]. For instance, studies have shown a close association between aging, neurodegenerative diseases, and impaired microglial function [4, 5]. In addition, abnormal increases in the numbers of CD4^+^ T cells, B cells, CD8^+^ T cells and dendritic cells (DCs) [6, 7] are involved in the pathogenesis and progression of MS. Other immune cells in the central nervous system (CNS), including boundary-associated macrophages (BAMs), have also been increasingly recognized in brain diseases [8]. These findings highlight the importance of investigating the immune microenvironment in the brain to understand the development of related brain diseases and design effective treatments or therapies [4–7].

**Fig. 1:**
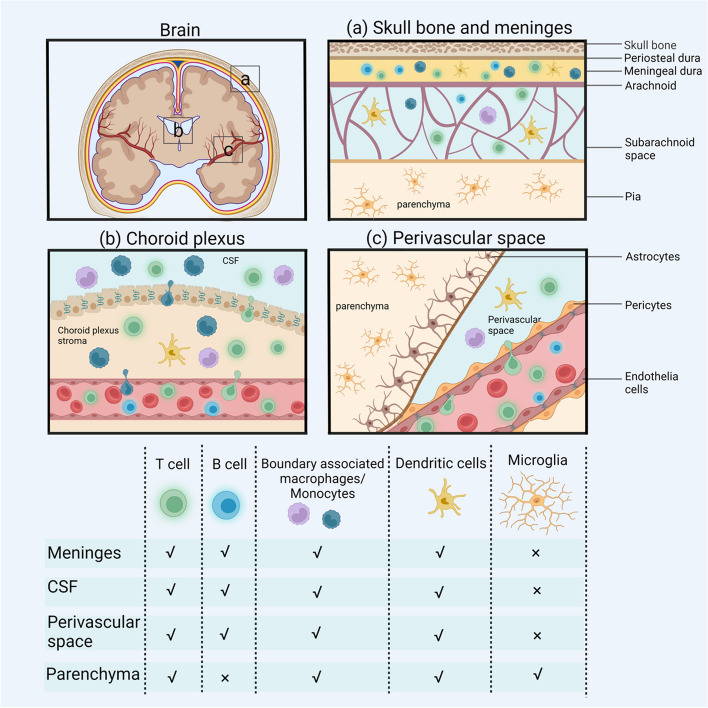
Main resident immune cells in the brain and their distribution in different brain regions. **a** Skull bone meninges. Lymphatic vessels shuttle through the dura mater, and immune cells in the dura mater are relatively numerous and abundant. The subarachnoid space is filled with CSF, which contains T cells, a small number of B cells, BAMs/monocytes and DCs. **b** Choroid plexus. Immune cells can enter the ChP matrix through fenestrated microvessels, but the tight junctions of the ChP basement membrane restrict immune cells in the ChP stroma from entering the CSF, decreasing the number and diversity of these cells in the CSF. **c** Perivascular space. A barrier formed by the endfeet of astrocytes separates the perivascular space from the brain parenchyma, with a small number and variety of immune cells present. Created with BioRender.com

The population and quantities of immune cells within the CNS differ among regions [9]. The distribution of immune cells in different regions of the brain is responsible for maintaining the homeostatic balance between brain structure and functions. In addition to the dura and pia membrane layers that cover the brain surface, the brain possesses several barriers: (i) the pia blood-cerebrospinal fluid (CSF) barrier, (ii) the endothelial BBB, (iii) the choroid plexus (ChP) and the blood-cerebrospinal fluid barrier (BCSFB) [10]. As shown in Fig. 1, the permeability of each barrier varies, and the entry of immune cells into CSF is limited by tight junctions (TJs) in the ChP basement membrane and brain microvascular endothelial cells. Normally, the pia meninges prevent the infiltration of patrolling immune cells into CSF, resulting in a decrease in the diversity and quantity of immune cells closer to the parenchyma [9]. Although the brain is rather sequestered, CSF interacts with the peripheral circulation and has advantages in terms of sample collection, and the ability to sample and analyze CSF may improve the diagnosis of brain disorders. Furthermore, the immune/inflammatory state of the brain may change before symptoms occur [11]. Studies have shown that the immune microenvironment changes during late–middle age prior to obvious signs of aging and the development of neurodegenerative diseases, suggesting that the immune microenvironment may be one of the causes of aging and neurodegenerative diseases. The role of immune cells, especially CD8^+^ T cells and B cells, as promising therapeutic targets warrants further investigation. However, the correlation between immune cell alterations in CSF and brain disorders has not been definitively established. In this study, we primarily reviewed the alterations in microglia, T cells, B cells, DCs, BAMs and other immune cells in the brain parenchyma, limbic space and CSF associated with aging, neurodegenerative diseases, MS, infectious diseases, and other conditions that affect the brain. We propose that the immune cell alterations within CSF have remarkable potential for predicting the onset and progression of brain disorders. Simultaneously, we summarize the potential origins and migration mechanisms of immune cells into CSF in both physiological and disordered states, aiming to enhance our understanding of these changes to improve the diagnosis and treatment of brain disorders.

### Alterations in immune cell subpopulation behind several brain disorders

#### Aging and neurodegenerative diseases

**Fig. 2:**
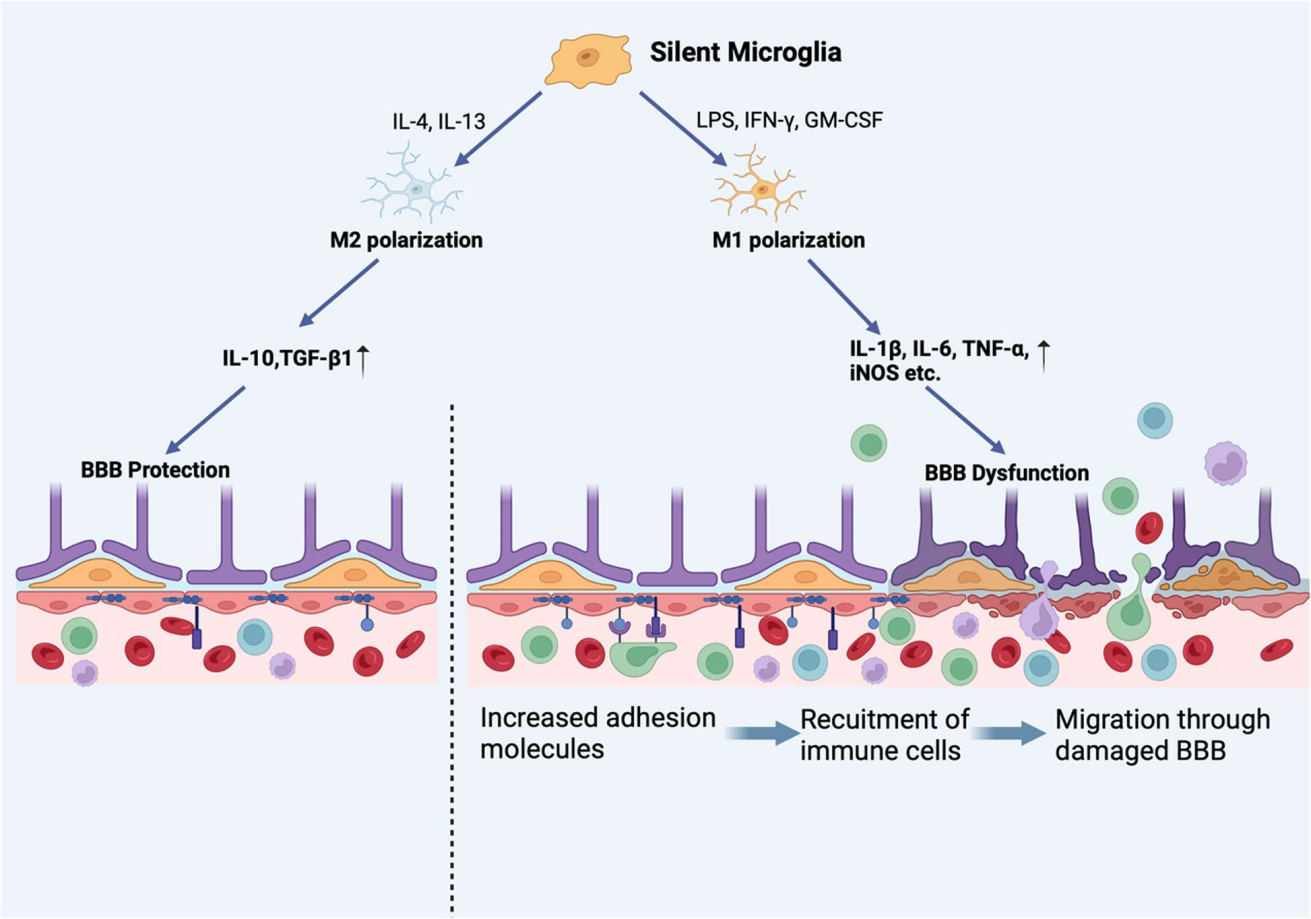
Dual effects of microglia on the BBB. Microglia can polarize into M1 and M2 subtypes under different stimuli and can exert destructive and protective effects on the BBB, respectively. Proinflammatory cytokines and iNOS upregulate the expression of adhesion molecules on endothelial cells and cause direct damage to the BBB (TJ, astrocyte endfeet and pericyte degradation), promoting the recruitment and migration of immune cells from the blood. Created with BioRender.com

Aging leads to the functional and structural degeneration of many organs, including the brain. Extensive research has been conducted to investigate the promotive effects of inflammation and immune microenvironment disorders on aging and the development of neurodegenerative diseases [2, 4, 12–16]. Microglia, the main resident immune cells in the brain, have been found to play an important role in aging and neurodegenerative diseases. Microglia have several functions in the brain, including executing immune surveillance against infiltrating immune cells, maintaining neuronal function and homeostasis, and activating other immune cell types [17, 18]. Microglia can differentiate into M1 and M2 subsets in response to different stimuli (this will be discussed later in the next section) [19]. M1 microglia induce inflammation and neurotoxicity, whereas M2 microglia exert anti-inflammatory and neuroprotective effects [20]. Thus, alterations in the quantity and function of microglial subsets can result in damage to the brain. Over time, microglia become hyperactivated, and the balance of microglial subsets shifts toward the M1 subset [21]. Moreover, aging neurons exhibit heightened responsiveness to Toll-like receptors (TLRs), which are expressed on neurons and glial cells [22] and are specific markers of the M1 subset, potentially leading to neuronal damage within the brain parenchyma through the activation of immune cell-mediated cytotoxicity and BBB dysfunction (Fig. 2) [2, 23]. In addition, the disruption of microglial sensing functions can initiate or exacerbate neurodegenerative diseases by increasing amyloid-β (Aβ) deposition [18]. The Aβ protein can in turn bind to TLR4 on microglia and trigger the secretion of inflammatory factors [24], which can lead to neuronal damage [19]. and impair the ability of microglia to clear Aβ [18, 25]. Moreover, hyperactivated microglia were found to improperly load pathogenic proteins onto major histocompatibility complex (MHC)-II molecules, initiating adaptive immune-mediated neuronal damages. [26]. In summary, as the most abundant immune cells in the brain, overactivated M1 microglia can promote aging and neurodegenerative diseases through direct neuronal damage, BBB impairment **(**Fig. 2**)** and Aβ deposition promotion.

In addition to microglia, T cells, B cells and monocytes have also been linked to the development of neurodegenerative diseases [2]. CD4^+^ T cells have been identified as mediators of neurodegeneration [26]. Furthermore, the oversecretion of IFN-γ by CD4^+^ T cells promotes the infiltration of circulating monocytes into the CNS and causes damage to peripheral neurons [27]. In addition, CD8^+^ T cells exert a substantial influence on CNS neurodegeneration through MHC-I-mediated activation and through their combined effects with CD4^+^ T cells [26]. Evidence has also shown increased IgG levels in the CSF of Parkinson's disease (PD) patients [28, 29]. and elevated B-cell infiltration in the brain parenchymas of AD patients, which promotes the deposition of immunoglobulin around Aβ plaques, suggesting a potential role of B cells in the development of neurodegenerative diseases [30].

#### MS

MS is an autoimmune disease of the CNS characterized by inflammation-induced demyelination, axonal loss and neuronal damage. The pathogenesis of MS is mediated mainly by myelin-reactive CD4^+^T helper (Th) cells [31]. Under steady-state conditions, the CNS is continuously monitored for damaging agents by T cells in CSF, but T-cell entry into the brain parenchyma is restricted by the BBB [32]. The infiltration of CD4^+^ T cells into the brain is considered a prominent feature of MS that precedes the onset of symptoms because the accumulation of CD4^+^ T cells damages the brain parenchyma and contributes to the development of MS. CD4^+^ T cells encompass multiple subsets, such as Th1, Th2, Th17, follicular T (Tfh) and regulatory T (Treg) cells, among which Th1 and Th17 cells play major roles in MS progression by secreting proinflammatory cytokines (*e.g., *IFN-γ) [33]. Evidence has suggested that Th1 cells can induce brain tissue damage by activating downstream microglial polarization to the M1 subset (which will be discussed later) [34]. In addition to Th cells, clonal expansion and activation of CD8^+^T cells have been observed in the CSF of MS patients [35, 36], and some of these cells persist in the CSF for a long time.  Moreover, the substantial clonal expansion of CD8^+^ T cells in CSF may be a sign of disease aggravation. However, the factors that promote the clonal proliferation of CD8^+^ T cells have not been fully elucidated [37]. Numerous studies have provided evidence of CD8^+^ T-cell activation and expansion in the CSF, suggesting that these cells contribute to tissue damage [38].

In addition to T cells, DCs and B cells are also involved in the pathogenesis of MS due to their role in antigen presentation [39]. Experimental studies have shown substantial DC infiltration into the brain during experimental allergic encephalomyelitis (EAE) [40]. Moreover, DCs enhance the severity of MS [7]. by activating Th17 cells [41]. Studies have shown that B cells are absent in normal CSF but accumulate in CSF when neuroinflammation occurs [42]. The presence of IgG oligoclonal bands produced by B cells in the brains of MS patients can serve as a diagnostic marker for MS and an indicator of disease severity. Moreover, antibody-secreting B cells can potentially serve as diagnostic markers for relapsing–remitting MS. Memory B cells have also been found to promote the progression of MS by mediating the proliferation of autoreactive CD4^+^ T cells in a human leukocyte antigen DR (HLA-DR)-dependent manner [43]. B cells in the peripheral immune system can migrate into the brain. Upon reactivation by CD8^+^ T cells in the brain, they further differentiate into plasma cells that secrete antibodies that damage neuronal myelin proteins, which may account for the presence of oligoclonal bands in CSF [44–48].

Furthermore, microglia and BAMs also play important roles in MS [49]. Microglia can serve as downstream targets of inflammatory factors and directly regulate immune responses [34]. Proinflammatory cytokines and ROS produced by M1 microglia can damage the BBB, which effectively promotes the migration of autoimmune cells into the brain. Previous studies have shown that BAMs have dual effects on MS [50]. On the one hand, they can promote the development of MS by secreting inflammatory factors and recruiting T lymphocytes to induce neurotoxicity in neurons; on the other hand, BAMs have also been found to alleviate MS symptoms by assisting axon regeneration, clearing inhibitory myelin debris and secreting neurotrophic factors.

#### Infectious diseases

Infectious diseases in the brain can trigger both inflammatory and immune responses. Leukocytes are recruited to the infected CNS through the action of innate immune molecules expressed by various brain cells (*e.g*., resident macrophages and microglia) [51]. This recruitment facilitates inflammatory responses and promotes pathogen clearance by multiple cell types. Although neuronal damage occurs during acute inflammatory responses, neurological dysfunction caused by infectious diseases may be primarily attributable to the long-term immune response mediated by T cells after pathogen clearance [51]. After the invasion of pathogens into the brain, microglia respond by recognizing pathogen-associated molecular patterns (PAMPs) on the pathogenic microorganisms through their pattern recognition receptors (PRRs) and produce death signals or activate inflammatory mediators such as interleukin 6 (IL-6) family cytokines [52]. This phenomenon is further supported by the finding that a reduction in the expression level of caspase-1, an inflammasome component that when activated leads to the release of IL-1β, has a protective effect on neurons [53]. The relative abundance and activation of microglia vary across different brain regions during nervous system infection [51]. The overactivation of microglia is conducive to local neurodegeneration and contributes to infection-related sequelae. Variations in the relative abundance and activation of microglia during the infection stage may aid in the identification of specific infection sites. However, whether and how microglia mediate infection in the brain remain unclear.

T cells have been implicated in the neurological dysfunction that arises from nervous system infection. Th1-biased cytokines, including IL-1β, tumor necrosis factor (TNF), and IFN-γ, may contribute to persistent cognitive impairment following bacterial meningitis [54]. However, the mechanisms underlying cognitive impairment during acute meningitis remain incompletely understood. It has been shown that neutrophils in the CSF of patients with tuberculous meningitis (TBM) are initially replaced by T and B cells during disease progression and persist for a long period after bacterial clearance [51]. We also observed a significant positive correlation between the quantity of T cells and the clinical severity of disease [55]. While Th1-polarized immune responses contribute to pathogen clearance, the production of IFN-γ by these cells also promotes the development of severe neurological diseases [51]. A comprehensive assessment of the function and antigen specificity of persistent T cells could lead to the establishment of new connections between microbial-specific immunity and psychiatric disorders. Importantly, T cells exhibit a sustained increase even after infection, and their levels correlate with the degree of neurological dysfunction after infection, suggesting that T cells may be new predictive markers for chronic neurodegenerative diseases caused by infection.

Pathogen-induced neuroinflammation can lead to a substantial increase in B cells within CSF [56, 57]. and B-cell accumulation in the periparenchymal and perivascular space [58]. B cells may play multifaceted roles in viral infection, as their protective function and involvement in antibody-mediated viral particle clearance have been demonstrated [59]. In progressive multifocal leukoencephalopathy caused by John Cunningham virus (JCV) infection, B cells not only act as a potential viral reservoir but also activate T cells by secreting cytokines and producing antibodies to promote virus clearance [60]. In conclusion, during the acute inflammatory phase of infectious brain diseases, the activation and recruitment of various immune cells are important for pathogen clearance.

### Other brain disorders

#### Glioblastoma (GBM)

GBM is one of the most common malignant primary tumors in the brain and is known for its high mortality and recurrence rates. Studies have shown that the immunosuppressive tumor microenvironment (TME) plays an important role in GBM treatment failure [61]. The formation of the TME is associated with the microglia, CD8^+^ T cell and DC dysfunction. Microglia promote the formation of the TME by secreting Th2-biased cytokines [62]. Furthermore, a special subset of microglia known as high-grade glioma-associated microglia (HGG-AM) has been identified as a mediator that shapes the cytokine microenvironment and promotes tumorigenesis [63]. These cells exhibit activated states and display proinflammatory and proliferative characteristics mediated by inflammasomes. GBM also impairs the integrity of the BBB, thereby facilitating the infiltration of CD8^+^ T cells into the CNS immune microenvironment. Conversely, the accumulation of regulatory T cells and suppressor immune cells in the brains of GBM patients suppresses the function of CD8^+^ T cells [64]. T-cell activation also occurs through the programmed death-1 receptor and its ligand (PD-1/PDL) pathway and through the cytotoxic T-lymphocyte-associated antigen 4 (CTLA-4) pathway [65]. In addition, although DC numbers are elevated in GBM patients, their antigen presentation capacity is attenuated. This inhibitory effect was found to be achieved by GBM cell-derived exosomal LGALS9 (lectin, galactoside-binding soluble 9), which targets DCs in CSF and suppresses the antitumor immunity of CD8^+^ T cells [66]. Taken together, these findings indicate that the accumulation of HGG-AM, regulatory and inhibitory immune cells, and exosomal LGALS9 in CSF may serve as markers for assessing the development and progression of GBM as well as a target for reversing its immunosuppressive TME status [64].

### Autism spectrum disorder (ASD)

The etiology of ASD is complex and not fully understood; however, genetic and environmental risk factors play a role [67, 68]. Studies have indicated that abnormal maternal immune activation (MIA), a common environmental risk factor, causes inflammation and oxidative stress in the placenta and fetal brain, resulting in neurodevelopmental impairments in the developing fetal brain and subsequently causing behavioral symptoms in offspring [69–71]. Inflammatory cytokines produced by maternal infection have persistent effects on fetal development by acting on the placenta or crossing the placenta into the fetal compartment [71–73]. For example, increased levels of IL-6 during pregnancy induced the secretion of IL-17a, which increased the IL-17 receptor levels in the brains of offspring, leading to ASD-related cortical and behavioral abnormalities [72, 74]. In addition, epidemiological and experimental studies have shown that maternal autoantibodies [75]. cross the placental barrier to recognize proteins in the developing fetal brain and affect the development of the nervous system. The ASD subtype resulting from this pathogenesis is termed maternal autoantibody-related (MAR) ASD. In addition to prenatal risk factors, immune dysfunction has also been observed in the brains of children with ASD [71]. In this case, immune dysfunction is manifested by a shift in cell subsets caused by increased inflammatory factors, including eotaxin, IL-6, IL-1 IFN-γ, macrophage inflammatory protein-1α (MIP-1α) and MIP-1 [76–78]. Additionally, there is an increase in baseline natural killer (NK) cell activity. Among these factors, the concentrations of MIP-1α and MIP-1β have been reported to be associated with social behavior disorders [77], and the IL-1β concentration has been reported to be positively correlated with social and nonverbal communication disorder behavior [78]. However, the causal relationship between these inflammatory factors and ASD has not been fully elucidated. Other diseases, such as anxiety-like behavior and acute injury, have also been found to be associated with brain immune status [79, 80], further underscoring its importance.

In summary, numerous brain disorders are accompanied by brain inflammation; however, the causal relationship remains unclear. Notably, there are common immune cell alterations that occur across different brain disorders. For example, in various brain disorders and inflammatory states, there is a prevalent pattern of microglial overactivation accompanied by increased numbers of key immune cell populations, such as CD4^+^ T cells, CD8^+^ T cells, B cells, DCs, and BAMs, in the brain (Table 1). Therefore, elucidating the mechanisms underlying these immune cell increases in inflammatory states is important for understanding the etiology of disorders in the brain and for developing therapeutic strategies.

**Table 1:** Changes in the number of immune cells in the brain of different diseases

Diseases	Cell types	Changes	Ref.
**Aging and Neurodegenerative Diseases**	Microglia	M1 increase M2 decrease	[21] [21]
	CD4^+^ T cells	Increase	[81]
	B cells	Increase	[30]
**MS**	CD4^+^ T cells	Increase	[31]
	CD8^+^ T cells	Increase	[35, 36]
	DCs	Increase	[40]
	B cells	Increase	[42]
**Infectious Diseases**	T cells	Increase in early stages and persist after pathogen clearance	[51]
	B cells	increase	[57]
**GBM**	DCs	Increased, with decreased activity	[66]
	CD8^+^ T cells	Increased, with decreased activity	[64]
	Tregs	Increased	[64]

## Identification of immune cells in CSF to elucidate brain immune changes

The number and functions of immune cells are dramatically altered in patients with various brain disorders. Moreover, the infiltration of peripheral immune cells into CSF and alterations in immunological homeostasis in the brain may occur before disease symptom onset, which suggests that the identification of such alterations may be used for early diagnosis [11]. In addition, analyzing CSF from patients at different stages may be an effective means to determine disease progression. Thus, several diagnostic methods for brain diseases based on immune cell changes in CSF have been proposed [82, 83], providing candidate targets for the early diagnosis and treatment of brain diseases.

### Microglia

Microglia are among the most abundant resident immune cells in the brain parenchyma. Recent studies have shown that microglia are derived from CD45^−^c-kit^+^ (c-kit proto-oncogene protein) red marrow progenitor cells generated from the yolk sac [84]. During the early stages of embryonic development, differentiated CD45^+^c-kit^−^CX3CR1^+^ microglial progenitors colonize the brain through the blood circulation [84, 85]. The process of microglial development and homeostasis is regulated by a variety of factors, including the transcription factor PU.1 (a hematopoietic cell-specific family transcription factor), unit-related transcription factor 1 (Runx1), interferon regulator factor 8 (IRF-8), colony-stimulating factor 1 receptor 1 (CSF1R), musculoaponeurotic fibrosarcoma oncogene homolog B (MAFB) and its ligands, and noncoding small RNAs (ncRNAs) [86–88]. After being recruited to the brain through the IL-34-CSF1Ra pathway, macrophages subsequently colonize the CNS compartment under the regulation of the short-range signals Lys phosphatidylcholine (LPC) and adenosine triphosphate (ATP), which are generated by neuronal apoptosis [89].

Under normal circumstances, microglia undergo self-renewal to maintain homeostasis, and this process does not involve peripheral cells [90, 91]. Although the mechanism of microglial self-renewal has not been fully elucidated, it has been suggested that microglia maintain longevity and renewal by competing for the trophic factor IL-34. [92]. Microglia polarize into two distinct functional subtypes via a variety of receptors and signaling pathways [34, 93]. The proinflammatory cytokines IFN-γ, TNF-α and GM-CSF, as well as LPS alone, can promote M1 polarization through the Toll-like receptor-4 (TLR-4)/nuclear factor (NF)-κB, Janus tyrosine kinase-signal transducer and activator of transcription 1 (JAK-STAT1)/STAT3 and members of the mitogen-activated protein kinase (MAPK)/NF-κB pathways. M2 polarization can be activated by Th2-biased cytokines such as IL-4 and IL-13. The activation of microglia is also associated with several other receptors, including CX3C chemokine receptor 1 (CX3CR1), triggering receptor expressed on myeloid cells 2 (TREM2), P2X7R and P2Y12R (two purinergic receptors in microglia) [94–97]. The CX3CR1-CX3CL1 pathway can inhibit the activation of microglia (M1) and the release of inflammatory factors upon lipopolysaccharide (LPS) treatment [98]. In an EAE model, elevated expression levels of CX3CR1 in M1 microglia suppressed their excessive activation. In the AD brain, TREM2 is involved in the overactivation of microglia [99]. Microglial proliferation, migration and cytokine secretion can be activated by Aβ, which is subsequently cleared by binding to TREM2 on the surface of microglia. Soluble TREM2 (sTREM2) was found in the CSF of patients with AD and MS [100]. P2X7R is regarded as a key molecule on the surface of the M1 subset and is involved in neuroinflammation and neurodegeneration [96]. However, P2Y12 was found to be downregulated in M1 microglia [101]. and to regulate the translocation of microglia in the brain, which ensures the patrolling of microglia in the brain [97].

### CD4^+^ T cells

Although T cells, especially memory CD4^+^ T cells [102, 103], are present in the CNS of both healthy individuals and individuals with noninflammatory diseases, their levels are significantly increased in the CSF of individuals with inflammatory diseases [104]. In the inflamed CNS, the expression of cell adhesion molecules (CAMs) and chemokines, including P-selectin, leukocyte function-associated antigen 1 (LFA-1), intercellular adhesion molecule 1 (ICMA-1), α4 integrin, chemokines and their receptors, such as atypical chemokine receptor 1 (ACKR1), are upregulated in vascular endothelial cells, facilitating the recruitment of CD4^+^T cells to the vascular endothelium to eventually cross the BBB [102, 104, 105]. Moreover, activated CD4^+^ T cells can cross the perivascular glial boundary and reach the parenchyma through a process mediated by activated matrix metalloproteinase (MMP)-2, MMP-9 and chemokines [10]. Current research has shown that a wide variety of surface receptors, including C–C chemokine receptor (CCR) 6, CCR2 and CCR4, are associated with the migration of CD4^+^ T cells [106]. Th17 cells can enter CSF through the ChP in a CCR6/CCL20 (C–C chemokine ligand 20)-dependent manner to initiate EAE [107]. After entry, cells independent of CCR6 may be recruited by chemokines released by Th17 cells that previously entered.

Additionally, c-Met^+^ (a tyrosine kinase receptor) CD4^+^ T cells are more susceptible to pathogenicity and migration due to their polarization toward proinflammatory phenotypes and upregulated expression of integrin α4 [108]. However, whether c-Met is merely a surface marker of T cells or plays a regulatory function and the pathway that regulates the production of CD4^+^ c-Met^+^ subsets remain unclear. Moreover, evidence suggests that aggressive T cells may be activated in the lungs before entering the brain and subsequently upregulate the expression of chemokine receptors and adhesion receptors with enhanced transferability [109], which may also enhance T-cell entry into the CNS and induce autoimmune disease.

### CD8^+^ T cells

Unlike that of CD4^+^ T cells, the migration of CD8^+^ T cells into the brain has not been fully elucidated, but their increased numbers and direct damage to neurons illustrate the need to elucidate their migration process. The migration of T cells at different stages is regulated by various chemokines and adhesion molecules. Previous studies have reported that the level of C-X-C chemokine ligand 13 (CXCL13) is significantly increased in CSF and can recruit CXCR5^+^ (C-X-C chemokine receptor type 5) CD8^+^ T cells to the brain [110]. Previous studies have also shown that antigen-specific clonal expansion of CD8^+^ memory T cells occurs in the inflamed brain and that the presence of antigens promotes the migration of CD8^+^ T cells [37]. These molecules may serve as targets for inhibiting inflammatory damage related to CD8^+^ T-cell migration in the brain.

### DCs

In brain inflammation, the number of myeloid and plasmacytoid DCs increases to varying degrees [111], and most of these DCs have been recruited from peripheral cells. The migration of DCs into the brain is affected by a variety of chemokines and their corresponding receptors [112]. Immature DCs expressing CCR2, CCR3 and CCR5, migrate to CSF and then drain out of CSF to cervical lymph nodes [40]. The increased migration of immature DCs to the CNS is mediated by a variety of inflammatory factors, such as TNF-α and IL-1β [113]. In mature DCs, CCR5 is not expressed, while CCR7 is upregulated [114]. The migration of mature DCs is mediated by the interaction of CCL19 and CCL21 with CCR7 and the CCL12/CXCR4 pathway [115]. Current studies report that immature DCs can interact more effectively with adhesion molecules such as α4β1 integrin expressed by inflamed endothelial cells, increasing the migratory potential of DCs to cross the vascular endothelium and enter the CNS [113]. Thus, immature DCs are more likely to be recruited into brain tissue to cross the BBB and then achieve maturation and promote antigen presentation in the brain [114]. In addition, the subpopulation of recruited DCs differs. CCR5 specifically recruits myeloid DCs (mDCs) to the CSF of patients with MS and acute optic neuritis but has no influence on plasmacytoid DCs [116], suggesting that mDCs may play specific functions in MS pathology. In addition to the increased recruitment of DCs, enhancing the antigen presentation capacity of DCs also contributes to inflammation in the brain. For instance, reduced nicotinamide adenine dinucleotide phosphate (NADPH) oxidase 2 (also known as CYBB/NOX2) in DCs regulates endocytic myelin oligodendrocyte protein (MOG) antigen processing and supports MOG antigen presentation to CD4^+^ T cells [117]. Based on the importance of DCs in T-cell activation and migration, we speculated that blocking the pathway related to the migration of immature DCs into the brain may be an effective means to inhibit MS [118]. Although the upstream mechanism of DC activation and migration has yet to be explored, the different chemotactic pathways related to mature DCs, immature DCs and other DCs are mediated by different chemokines, indicating that these factors could be used to determine disease stage and type.

### B cells

B cells have been found in multiple regions of the CNS in MS patients, including the meninges, lesioned and normal white matter, the cortex, and CSF [119]. Although the distribution of B-cell clones in different brain compartments provides evidence that B cells can cross different CNS barriers, the mechanisms underlying how and where B cells cross these barriers have not been fully elucidated [47]. B-cell recruitment depends on a combination of adhesion-dependent signals and cytokines in the brain, such as very late antigen (VLA)-4, α4β1 integrin, CD44, CXCL13, CXCL12/SDF-1, IL-6, and TNF-α [47]. Among these chemokines, CXCL13 and VLA-4 play important roles. Blocking VLA-4 was found to reduce the number of B cells crossing the CNS barrier, whereas antibodies against vascular cell adhesion molecule 1 (VCAM-1, a ligand of VLA-4) did not [120, 121], indicating that the binding of other alternative CAMs to VLA-4 may be involved in B-cell migration. Moreover, the time-dependent changes in chemokines in the brain may influence the subsets of B cells that enter the CNS at different stages of disease, as B cells display distinct chemotactic signatures in the inflammatory state of the CNS [60]. Studies have shown that B cells in the meninges likely originate from the skull and reach the meninges from the skull through specialized vascular connections, suggesting that the meninges play an important role in the recruitment of B cells [122, 123].

### BAMs

Macrophages present in brain boundary regions, known as BAMs, originate from the same primitive macrophages produced by red myeloid progenitor cells in the yolk sac as microglia and are disseminated to the brain in the early stage of embryonic development [124]. Thus, receptors that mediate the development and migration of BAMs largely overlap with those of microglia and include PU.1, IRF8 and CX3CR1 [125]. However, recent studies have shown that only meningeal macrophages and microglia have the same prenatal progenitor cell, while perivascular macrophages originate from meningeal macrophages [126]. CCR1, CCR2 and CCR5 mediate the migration of macrophages to sites of inflammation, but the specific underlying mechanisms have not been elucidated and require further investigation [127].

Moreover, BAMs can be divided into dura, subdural, perivascular and choroid plexus macrophages according to their location. [8]. Recent studies have shown that BAMs have different regeneration capacities and prominent population heterogeneity according to their location [8, 125]. Dural and choroid plexus macrophages are partially dependent on the recruitment of peripheral blood monocytes, while subdural macrophages are rarely replaced by monocytes [128]. Thus, macrophages in the dura and choroid plexus have shorter life spans and are renewed more frequently. Two subgroups of BAMs, MHCII^lo^ and MHCII^hi^, were identified and characterized by different molecular characteristics. The former can be found in the dura and subdura, while the latter is found only in the dura [8]. The MHCII^lo^ subset has been suggested to have a negative regulatory effect on the immune response [128], while the MHCII^hi^ subset may be involved in immune monitoring in the dura [8]. Overall, the heterogeneity of BAMs in different regions needs to be fully elucidated to determine their contribution to brain diseases.

### Other immune cells

In addition to the abovementioned immune cells, several other cells, including NK cells and astrocytes, also affect immune homeostasis in the CNS. Studies have shown that NK cells exert multiple effects on the CNS [129]. NK cells are characterized by their ability to destroy targeted cells after being recruited by the CX3CL1/CX3CR1 pathway [129]. Therefore, the accumulation of NK cells in the nervous system increases the risk of developing autoimmune inflammatory diseases. In contrast, NKs can inhibit the development of autoimmune diseases in the brain, such as MS, as evidenced by decreased NK cell activity and number in the peripheral blood of MS patients [129, 130]. However, the mechanism by which NK cells suppress autoreactive diseases is not fully understood. Several studies have shown that NK cells can cause T-cell apoptosis by producing IFN-γ [131]. The harmful effect of NK cells is mediated by their ability to damage peripheral nerve cells through bystander effects, providing evidence that NKs promote the development of neurodegenerative diseases [132]. Additionally, IFN-γ secreted by NK cells enhances the antigen presentation ability of APCs by increasing the expression of MHC class II molecules, which promotes the activation of T cells and enhances the inflammatory response [129].

Future studies should also address the interactions of other cells with different subsets of astroglia cells and find ways to stabilize and control their subsets and functional balance, which is highly important for the treatment of neurological diseases.

In conclusion, due to the continuous excavation of the complex structure and cellular function of the brain, an increasing number of immune cells has been found to have an impact on brain diseases. For example, a novel glial progenitor cell (GPC) was recently identified to have both astrocyte and oligodendrocyte gene expression signatures [133]. This study provides a framework for future studies on the function of these two cell types. However, there are still other unidentified immune cells that play a role in brain disease. Moreover, due to the heterogeneity of brain immune cells and immune factors as a result of different regions and sources, clarifying and isolating the multiple functions of known immune cells is also an important direction for future research. Elucidating these scientific mysteries may lead to new strategies for the precise targeted therapy of neurological diseases.

## Targeting immune cells for the diagnosis and treatment of brain diseases

The immune microenvironment in the brain is altered in the early stage of many diseases, even before the onset of symptoms. Excessive inflammation in the brain causes nerve damage and functional disorders. Based on the advantages of identifying early changes in the immune microenvironment and the sampling methods mentioned above, we focused on immune cell and immunomodulatory factor alterations in CSF, which may be beneficial for the early diagnosis of neurological diseases. We also summarized the current treatment methods and potential therapeutic targets for the brain disorders mentioned above.

### Diagnosis

Many immune cells, factors and chemokines are significantly increased in the CSF of patients with various brain diseases **(**Tables 1, 2**)**. For example, quantifying B-cell numbers in CSF may be a potential strategy for diagnosing MS, as B-cell clone amplification is a common characteristic of the disease [45]. A diagnostic method based on the monocyte/lymphocyte ratio has been proposed to predict cognitive conditions in aging and neurodegenerative diseases [82]. IgG oligoclonal bands in CSF have also been used to diagnose MS [83]. However, immune diagnosis based on changes in immune cells is currently used as an auxiliary diagnostic method and not as a standard diagnostic therapy because the specific roles of these cells in different diseases have not yet been confirmed. Further elucidation of the contribution of each immune cell type to different diseases may help to improve the specificity of such diagnostic methods.

**Table 2:** Chemokines and receptors involved in the migration of immune cells in the brain

**Chemokines**	**Microglia** [134]	**Lymphocytes** [107, 110, 135]	**DCs** [7, 114, 136, 137]	**B cells** [47, 138]	**BAMs** [27]
IL-34^i^	CSF1R				
CCL20^m^		CCR6			
CCL2^i^		CCR2	CCR2		CCR2
CXCL13^i^		CXCR5		CXCR5	
CXCL12^i^		CXCR4		CXCR4	
CCL3^i^			CCR3		
CCL5^m^			CCR5		
CCL9/CCL21^i^			CCR7		

^I^ increase in inflammatory state

^m^ increase first and then decrease in inflammatory state

Receptors are expressed on the corresponding immune cells

Although T-cell infiltration in the early stage is present in MS patients and mouse models of EAE, the current diagnosis of MS relies mainly on magnetic resonance imaging (MRI) data combined with the increased levels of oligoclonal IgG and monocytes in CSF [139, 140]. Early diagnosis strategies based on T cells have not yet been established. Importantly, the accumulation of cells characterized by the expression of TNF-α, IFN-γ, IL-2, CXCR4 and VLA4 in the blood of MS patients also provides candidate targets for the early diagnosis of MS [34].

### Treatment targets

#### Microglia

**Fig. 3:**
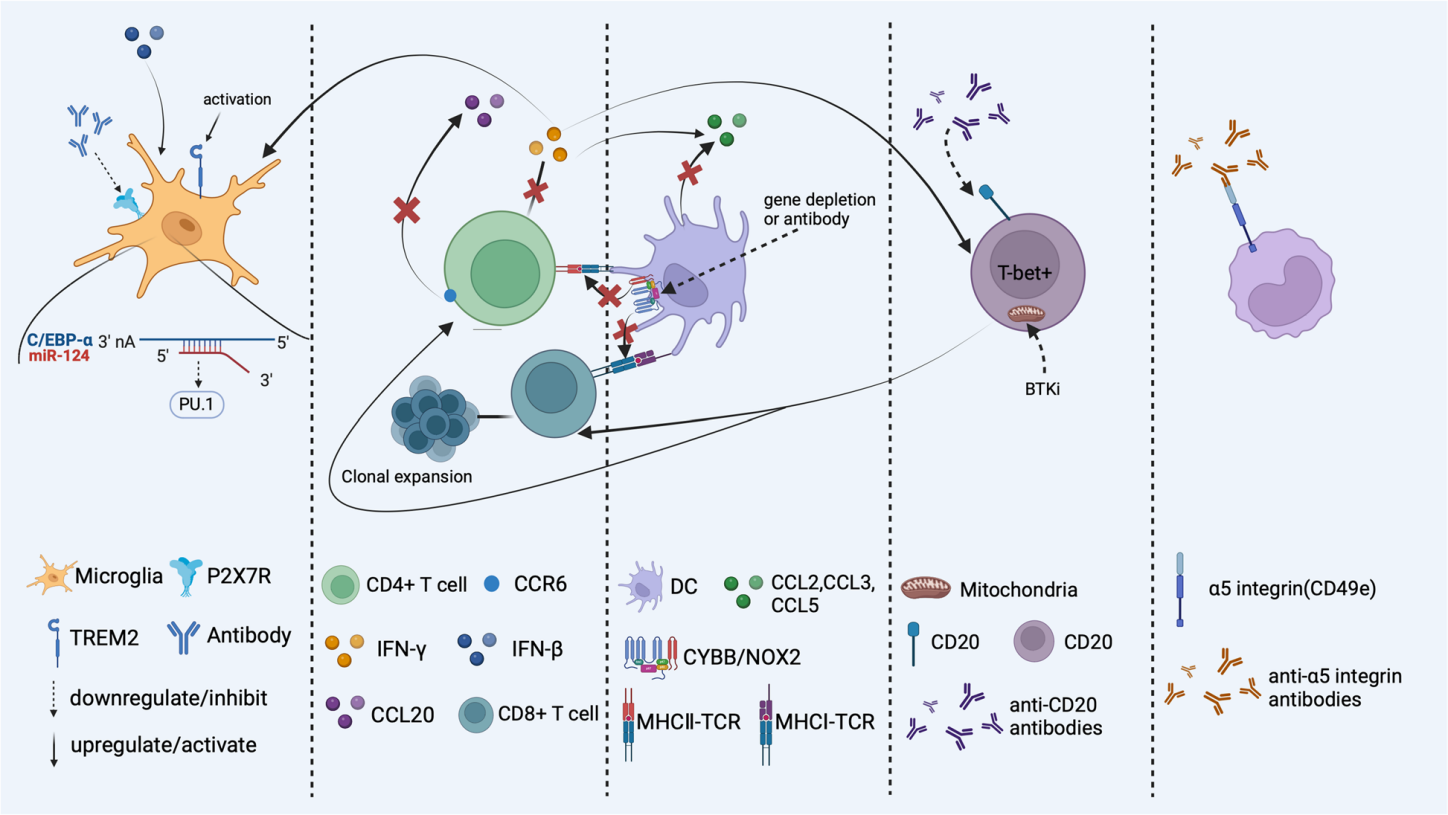
Therapeutic targets for brain disorders Inhibition of P2X7R and activation of TREM2 can promote the transition of microglia to the M2 subtype. miRNA-124 inhibits PU.1-induced microglial proliferation by reducing the synthesis of C/EBP-α. CCR2 is a target for reducing the recruitment of CD4^+^ T cells to the brain. IFN-γ secreted by CD4 + T cells can induce the activation of the M1 subset, while IFN-β promotes the activation of M2 and B cells and increases the levels of CCL3 and CCL5, which are responsible for immature DC recruitment. CYBB/NOX2, which can improve the antigen presentation capability of DCs, is another target for an excessive immune response and can be inhibited by gene depletion or antibody neutralization. Anti-CD20 antibodies can reduce the number of T-bet^+^ B cells induced by IFN-γ. BTKis can also inhibit the function of B cells by inhibiting mitochondrial respiration and thus reduce the activation of T cells. The α5 integrin is a target for reducing the recruitment and infiltration of monocytes in the CNS. Created with BioRender.com

As illustrated above, the balance of microglial polarization is disrupted in the inflamed brain. M1-type microglia secrete pro-inflammatory cytokines and neurotoxic mediators, which worsen neuronal damage. On the other hand, M2-type microglia promote an anti-inflammatory response that aids in repairing. It is crucial to finely regulate the activation of M1 and M2 microglia to minimize damage and maximize protection, as this has significant therapeutic potential. Currently, the main treatment strategy is to inhibit the signaling pathway of M1 microglia and promote the transformation of the M1 subtype to the M2 subtype (Fig. 3). Therefore, reducing the levels of IFN-γ and IFN-β is a potential way to alleviate inflammation in the brain [93]. In addition, antibody neutralization and microRNA (miRNA) interference are major means to specifically inhibit microglial activation and proliferation. miR-124, a highly expressed noncoding small RNA in quiescent microglia, can slow the progression of EAE by promoting microglial quiescence [141]. In addition, promoting Aβ clearance induced by activated TREM2 provides new insight into AD treatment [99]. Moreover, TREM2 has also been found to play an anti-inflammatory role in EAE, but its specific mechanism has not been fully elucidated [98]. Based on the role of P2X7R in microglial development [96]. genetic depletion or pharmacological inhibition of P2X7R was found to ameliorate the symptoms of AD in model mice [142]. However, its effectiveness needs to be further verified in AD patients.

### T cells

Since CD4^+^ T cells play a major role in MS, reducing the recruitment of overactivated CD4^+^ T cells to the brain is an important strategy for alleviating MS. Moreover, as adhesion molecules help T cells and other immune cells migrate to the brain [105], targeting the inhibition of adhesion molecules may be a new therapy [143]. In addition, chemokine receptors related to T-cell migration, such as CCR6 and CCR2 (Fig. 3), can also be targeted to reduce T-cell infiltration in the brain [107, 108, 144]. Inflammatory factors secreted by T cells, such as IFN-γ (Fig. 3), TNF-α and IL-1β, also affect the infiltration and function of other immune cells, suggesting the importance of suppressing T-cell hyperactivation [113, 145–148]. In addition to traditional antibody targeting and gene silencing, current studies have shown that nanomedicine also exhibits a suitable inhibitory effect on the expression of inflammatory factors [149]. However, IFN-γ has been shown to indirectly aid in the early treatment of EAE [150], which is contradictory to the known effects of IFN-γ, and the mechanism underlying the regulation or inhibition of neuroinflammation needs to be further explored. Based on the hypothesis that clonal proliferation of CD8^+^ T cells in MS may be associated with EBV infection [37], preventing viral infection or peripheral escape may also be effective at inhibiting the onset of MS. Recent studies have shown that ferroptosis is an early event in EAE and can promote T-cell activation in vitro and in vivo; thus, ferroptosis is a potential therapeutic target for MS [151].

#### DCs

Strategies for eliminating DCs include reducing the recruitment of peripheral DCs, especially immature DCs, to the brain and reducing the antigen-presenting ability of DCs. Depletion of chemokines has been shown to reduce DC recruitment to the brain and slow the progression of EAE [115] (Fig. 3). Moreover, genetic depletion of Cybb in cDCs has also been shown to reduce the excessive entry of T cells into the CNS and thus slow the progression of EAE [117] (Fig. 3).

#### B cells

In response to immunoglobulin production and B-cell lineage expansion in the CSF of MS patients, B-cell antigen-driven maturation and B-cell depletion therapy have been used to treat MS [152]. Antibody-induced depletion of B cells has been found to be effective at alleviating disease symptoms and has gradually attracted increasing amounts of attention [30, 47, 153]. Bruton's tyrosine kinase (BTK) is an important downstream molecule of B-cell receptors. BTK inhibitor (BTKi) has been found to limit the proinflammatory activation of B cells and T cells by inhibiting mitochondrial respiration in B cells [154] **(**Fig. 3**).** The enrichment of CD20 on IFN-γ-induced T-bet-expressing IgG^+^ B cells indicates that T-bet-expressing IgG^+^ T-cell subsets are important therapeutic targets [6].

#### Monocytes/BAMs

Because the number of monocytes recruited to the brain increases significantly under inflammatory conditions, reducing the number of migration receptors on the surface of monocytes is a promising inhibitory effect. CD49e, an α5 integrin, is specifically expressed on peripheral monocytes but not on the resident myeloid cell population. Treatment with the anti-CD40e antibody blocked the migration of peripheral monocytes into the brain and significantly slowed the progression and severity of EAE [155]. In addition, transient depletion or suppression of Tregs facilitates monocyte/macrophage recruitment to the brain and the clearance of amyloid, which further ameliorates neuroinflammation and reverses cognitive decline [156].

In summary, most current therapies tend to inhibit B cells and target receptors and factors that cause the abnormal activation, proliferation, or recruitment of immune cells to the treatment site using antibodies. The method of silencing genes through miRNAs has also been used [157]. However, the specificity and efficacy of these targets cannot be guaranteed at present.

Overall, the source and specific pathway through which immune cells enter the CNS have not been fully elucidated, and the contributions of different immune cell abnormalities to neurodegenerative diseases is also unclear. Moreover, whether treatment against these immune targets causes other side effects remains to be explored. More work is needed to address these knowledge gaps and to improve the diagnostic specificity and treatment efficacy.

## Conclusions

The immune microenvironment plays an important role in maintaining the normal physiological functions of the brain. As outlined in the Introduction, even under physiological conditions, the distribution of immune cells in the brain is not homogeneous because of the regions formed by barriers. The CSF can provide fundamental information about the inflammatory processes circumscribed to the CNS and reflects changes in the immunological pattern due to the progression of the pathology, and hence be used as a relatively non-invasive liquid biopsy allowing the most accurate measurement of the degree of blood-CSF barrier permeability. The abnormalities in the number, function, location and the excessive activation state of immune cells in CSF, including those in microglia, T cells, B cells, DCs, and BAMs/monocytes cause the promoted inflammatory state of the brain, resulting in direct or indirect damage to neurons and facilitating the progression of symptoms in a variety of brain diseases. These findings highlight the utility of measuring CSF immune changes to identify disease-associated neuroinflammation and to reflect the pathobiological events of brain disorders.

Moreover, because the analysis of immune cells of the CSF can provide a non-invasive alternative to predict the onset of brain disorders, immune cells appear to be potential targets for the diagnosis and treatment of certain diseases. Several researchers have proposed that brain diseases can be predicted or diagnosed by determining the number or fraction of immune cells in CSF. Although further research is needed to be performed to enhance the specificity and sensitivity of these indices, CSF immunophenotyping may be useful to gain further insight into the regulator cells and cytokines involved in the pathophysiology of barrier and function impairment. Treatment-related therapies targeting Tregs, B cells, and DCs have shown some efficacy in animal models, inspiring investigations to identify immune cells that could be new diagnostic and therapeutic targets, and to elucidate the unknown mechanisms and significance underlying immune cell migration, cell proliferation and activation in the brain microenvironment.

## Data Availability

Not applicable.

## Abbreviations

CSF: Cerebrospinal fluid
DCs: Dendritic cells
MS: Multiple sclerosis
BAMs: Boundary-associated macrophages
BBB: Blood-brain barrier
ChP: Choroid plexus
BCSFB: Blood-cerebrospinal fluid barrier
CNS: Central nerves system
TJ: Tight junction
TLRs: Toll-like receptors
JAK-STAT: Janus tyrosine Kinase- Signal Transducer and Activator of Transcription
IFN-γ: Interferon gamma
Aβ: Amyloid-β
AD: Alzheimer's disease
MHC II: Major Histocompatibility Complex II
PD: Parkinson's Disease
Th: T helper cells
Tfh: Follicular T cells
Treg: Regulatory T cells
EAE: Experimental allergic encephalomyelitis
HLA-DR: Human leukocyte Antigen DR
EBV: Epstein-Barr-Virus
PAMPs: Pathogen-associated molecular patterns
PRRs: Pattern recognition receptors
L-6: Interleukin 6
TNF: Tumor necrosis factor
TBM: Tuberculous meningitis
JCV: John Cunningham virus
GBM: Glioblastoma
TME: Tumor microenvironment
HGG-AM: High-grade glioma-associated microglia
PD-1/ PDL: Programmed death-1 receptor and its ligand
CTLA-4: Cytotoxic T-lymphocyte-associated antigen 4
LGALS9: Lectin, Galactoside-Binding Soluble 9
ASD: Autism Spectrum Disorder
MAR: Maternal autoantibody-related
NK: Natural killer cells
c-kit: C-kit: proto-oncogene protein
PU.1: A hematopoietic cell–specific ets family transcription factor
Runx1: Unt-related transcription factor 1
IRF-8: Interferon regulator factor 8
CSF1R: Colony-stimulating factor 1 receptor 1
MAFB: Musculoaponeurotic fibrosarcoma oncogene homolog B
NcRNAs: Non-coding small RNAs
LPC: Lys phosphatidylcholine
ATP: Adenosine triphosphate
Msr1: Macrophage scavenger receptor 1
DAMPs: Damage associated molecular patterns
Mafba and Mafbb: Analogues of mammalian transcription factor MAFB in zebrafish
Gpr34a: G-protein-coupled receptor 34a
CX3CR1: CX3C chemokine receptor1
TREM2: Triggering receptor expressed on myeloid cells 2
LPS: Lipopolysaccharide
P2X7R, P2Y12R: Purinergic receptor in microglia
sTREM2: Soluble TREM2
CAMs: Cell adhesion molecules
LFA: Leukocyte Function-associated Antigen
ICMA-1: Intercellular adhesion molecule-1
ACKR1: Atypical Chemokine Receptor 1
MMP: Matrix metalloproteinase
CCR: C-C chemokine receptor
CCL: C-C chemokine ligand
c-Met: A tyrosine kinase receptor
CXCR5: C-X-C chemokine receptor type 5
APCs: Antigen presenting cells
mDC: Myeloid DCs
NADPH: Nicotinamide adenine dinucleotide phosphate
CYBB/NOX2: Nicotinamide adenine dinucleotide phosphate oxidase 2
MOG: Myelin oligodendrocyte protein
VLA: Very late antigen
GPC: Glial progenitor cell
MRI: Magnetic Resonance Imaging
miRNA: Micro-RNA
BTK: Bruton's Tyrosine Kinase
BTKi: BTK inhibitor
